# Supplementary material for: Pm57 from Aegilops searsii encodes a tandem kinase protein and confers wheat powdery mildew resistance
Source: Nat Commun. 2024 Jun 5;15:4796. doi: 10.1038/s41467-024-49257-2 (PMC11153570; doi:10.1038/s41467-024-49257-2)
Supplement: Supplementary file 1 — Supplementary Information [file 41467_2024_49257_MOESM1_ESM.pdf]

_Pm57_ from _Aegilops searsii_ encodes a tandem kinase protein and confers wheat powdery mildew resistance

Zhao *et al.*

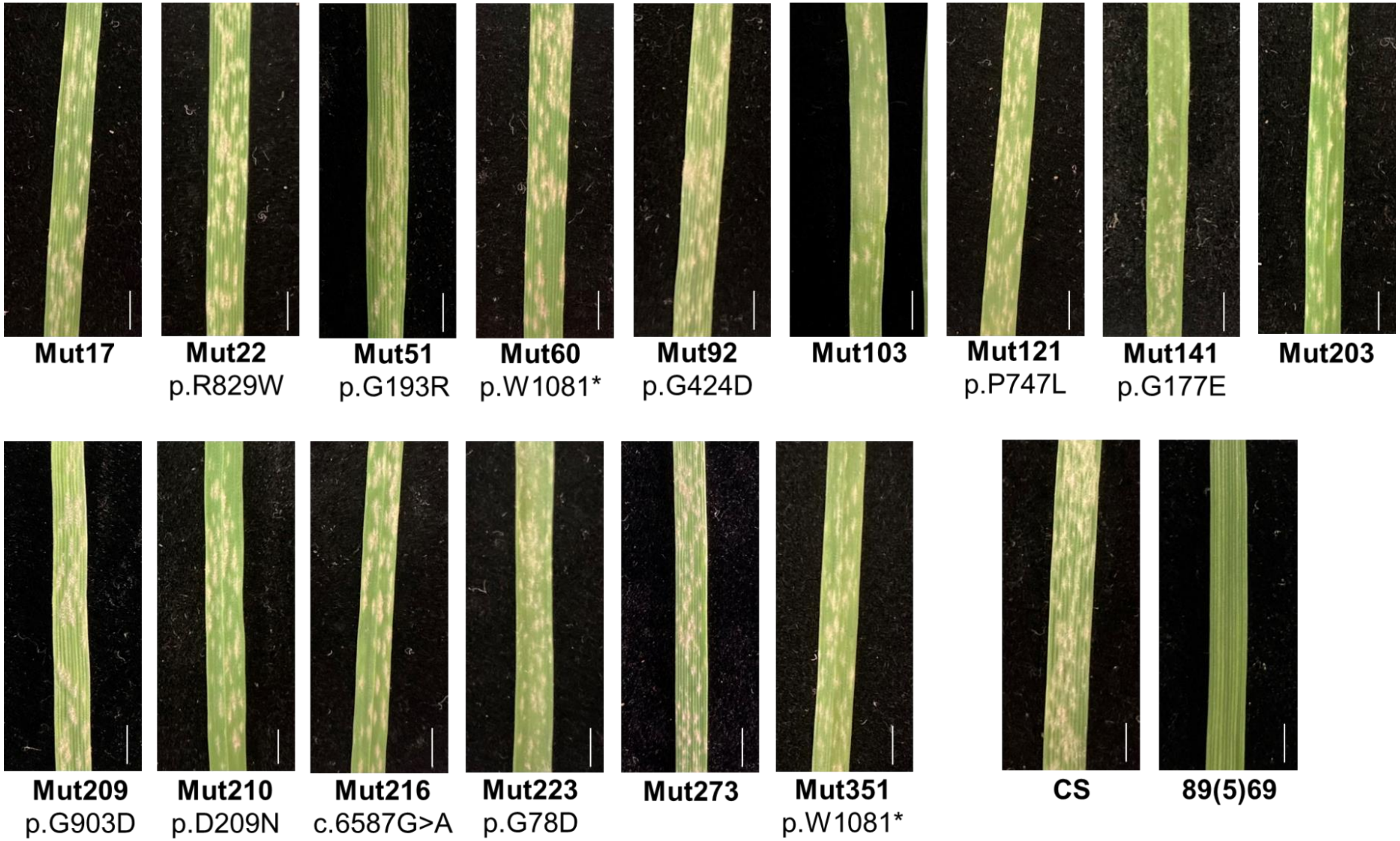

Supplementary Fig. 1. Powdery mildew resistance phenotypes of 15 EMS-induced susceptible mutants from CS-*Ae. searsii Pm57* introgression line 89(5)69. Images show a representative phenotype of the first leaf at 7 days post inoculation (dpi) with *Bgt* isolate E09 when the first leaf of each seedling had fully unfolded. Chinese Spring (CS, IT = 4) and the parental line 89(5)69 (IT = 0) were used as susceptible and resistant controls, respectively. Scale bars = 0.5 cm. The *G4* sequence variations in the susceptible mutants were indicated, and no sequence variations were found in Mut17, Mut103, Mut203, and Mut273.

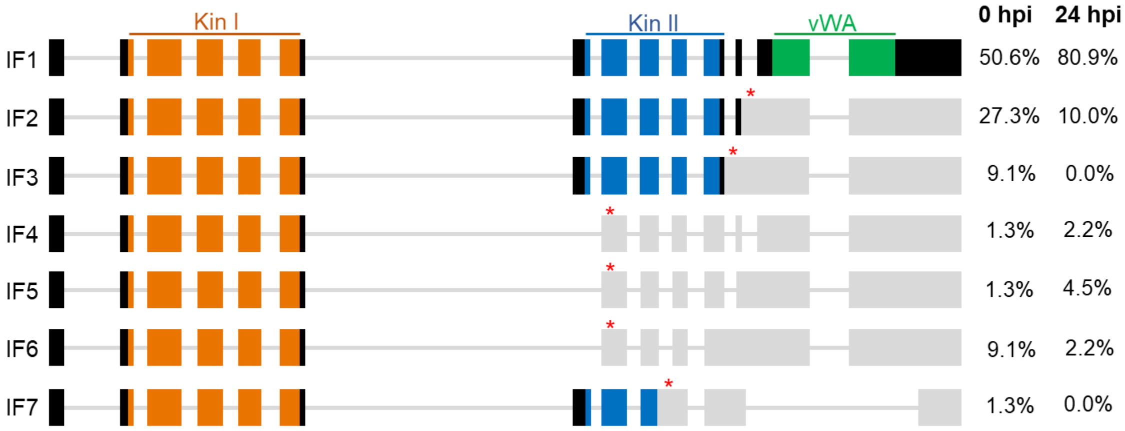

Supplementary Fig. 2. Schematic representation of *G4* alternative transcript variants. Exons and introns are represented by rectangles and lines, respectively. The two protein kinase domains and the vWA domain are shown in orange, blue, and green colors, respectively. The proportion of each isoform (IF) in the expression data of 89(5)69 at 0 and 24 h post inoculation (hpi) with *Bgt* isolate E09 is presented on the right side. IF1 is the main variant with 14 exons encoding for a complete protein. IF2-7 transcripts were derived from the mis-splicing of exon or intron and generated premature stop codon (marked in red star).

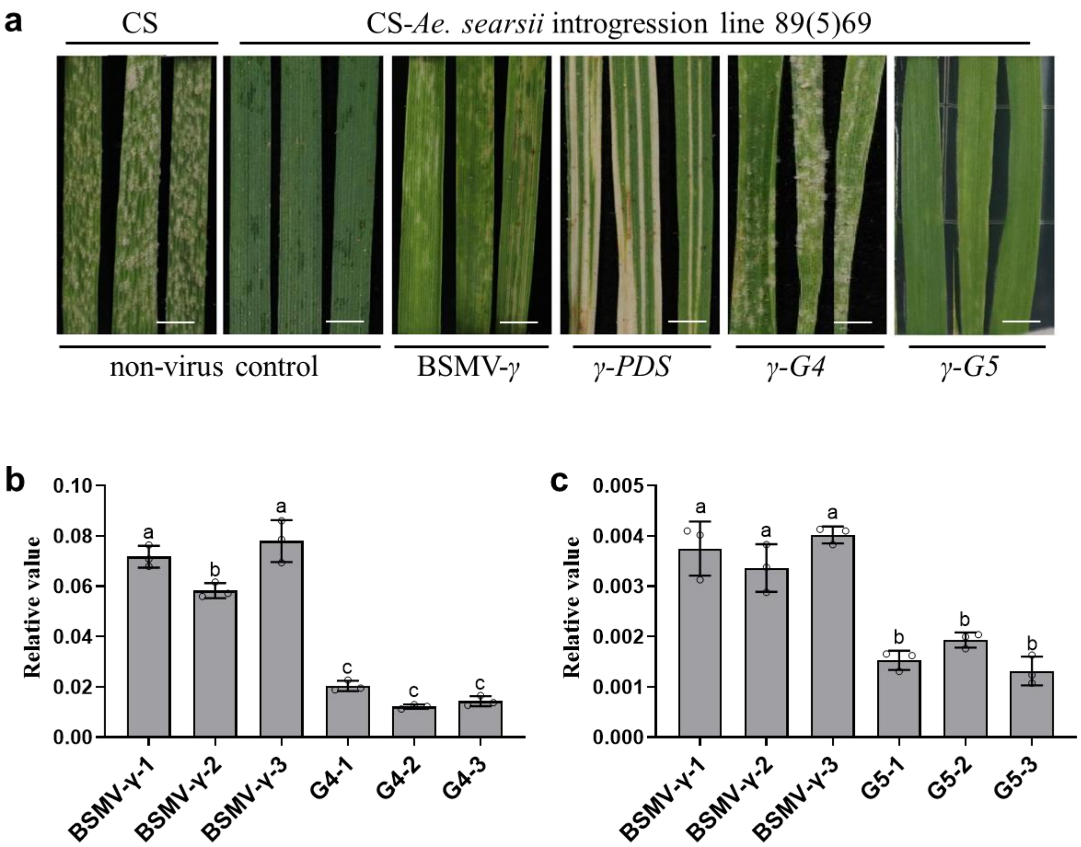

Supplementary Fig. 3. BSMV-VIGS mediated functional analysis of *G4* and *G5*. (**a**) Representative images showing the results of VIGS. γ-G4 (G4-VIGS) and γ-G5 (G5-VIGS) are two constructs targeting *G4* and *G5*, respectively. γ-PDS (BSMV-TaPDS) targeting phytoene desaturase gene (*PDS*) was used as a control to illustrate the effect of gene silencing, while BSMV-γ was used as the empty vector control. Images show a representative phenotype of the 3rd systemic leaf at 7 dpi from seedlings infected with *Bgt* isolate E09. Scale bars = 0.5 cm. (**b-c**) Expression levels of *G4* and *G5* in plants showing virus-infected phenotype. Relative expression levels of *G4* and *G5* in the 3rd and 4th leaves at 14 days after virus infection were determined by qRT-PCR. The 3rd and 4th leaves of each plant were mixed as one sample. qRT-PCR was performed three times for each sample, and each time as a technical replicate. The wheat *TaActin* was used as endogenous control, and the relative expression levels were calculated using the comparative CT method. Values represent means ± SD of three technical replicates. Different lowercase letters above the bars denote significant differences at the $p < 0.05$ level (One-way ANOVA). *G4* was efficiently silenced in infected leaves of 89(5)69 individuals (presented by No. 1, 2, 3), while they were normally expressed in BSMV:γ infected leaves. Source data are provided as a Source Data file.

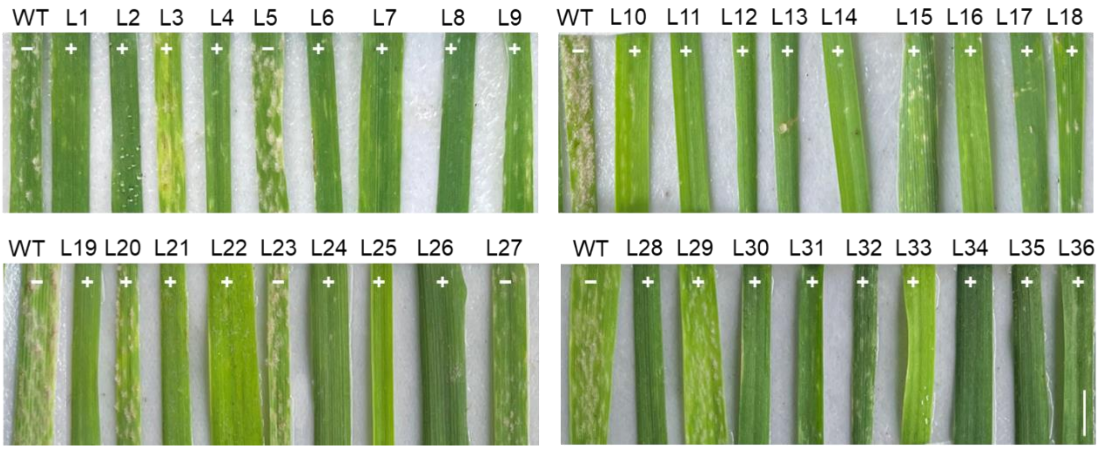

Supplementary Fig. 4. Responses of 36 independent T_0 transgenic plants (L1 to L36) to *Bgt* isolate E09. All the positive T_0 transgenic plants (+) excluding L20 and L29 were highly resistant to the *Bgt* isolate E09, whereas all the negative plants (−, L5, L23 and L27) were as susceptible as the WT Fielder. The '+' and '−' signs on each leaf designate the presence or absence of *G4* gene. Scale bars = 0.5 cm. Source data are provided as a Source Data file.

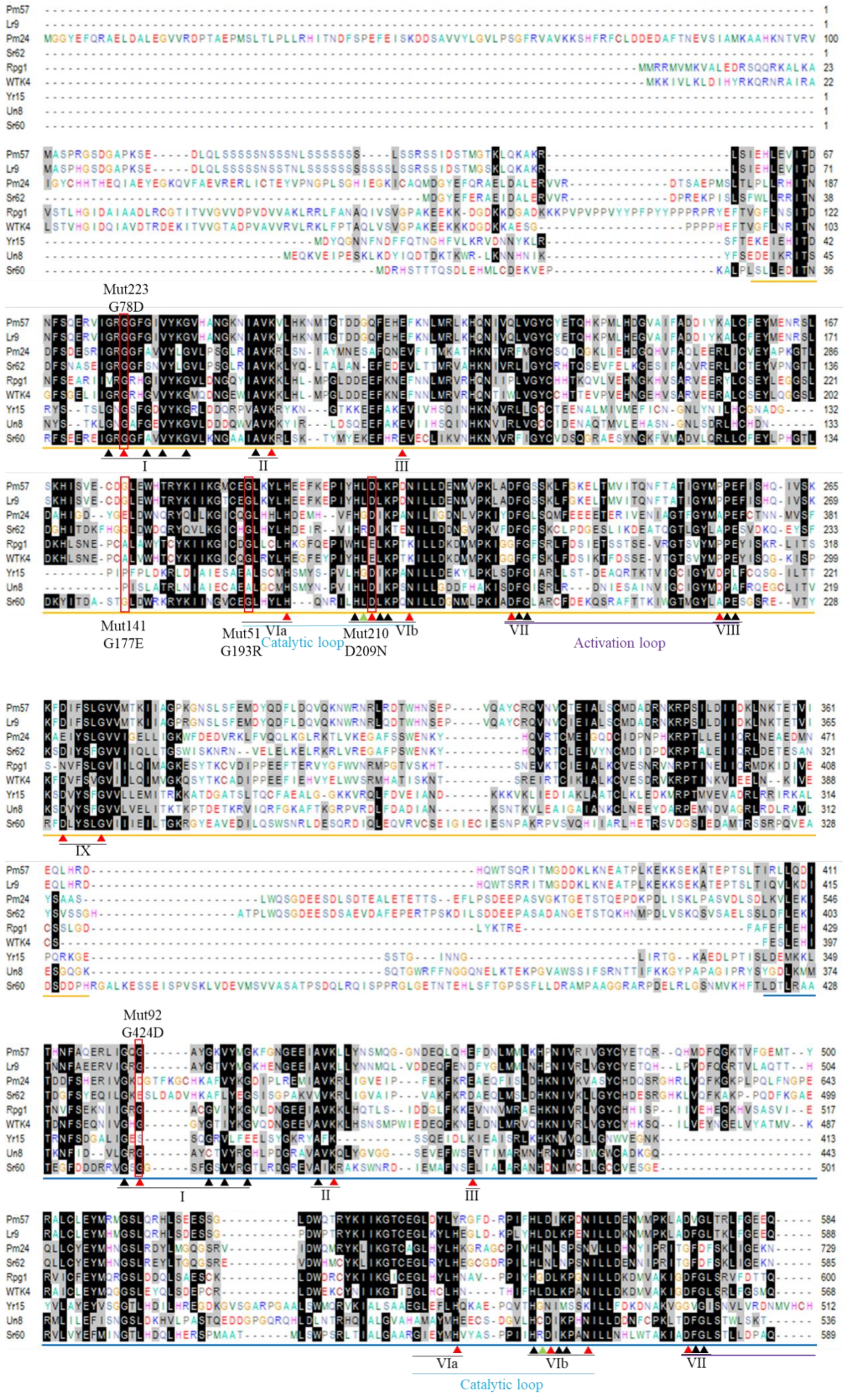

Pm57
Lr9
Pm24
Sr62
Rpg1
WTK4
Yr15
Un8
Sr60
Mut223
G78D
I
II
III
Mut141
G177E
Mut51
G193R
VIa
Catalytic loop
Mut210
D209N
VIb
VII
Activation loop
VIII
IX
Mut92
G424D
I
II
III
VIa
Catalytic loop
VIb
VII

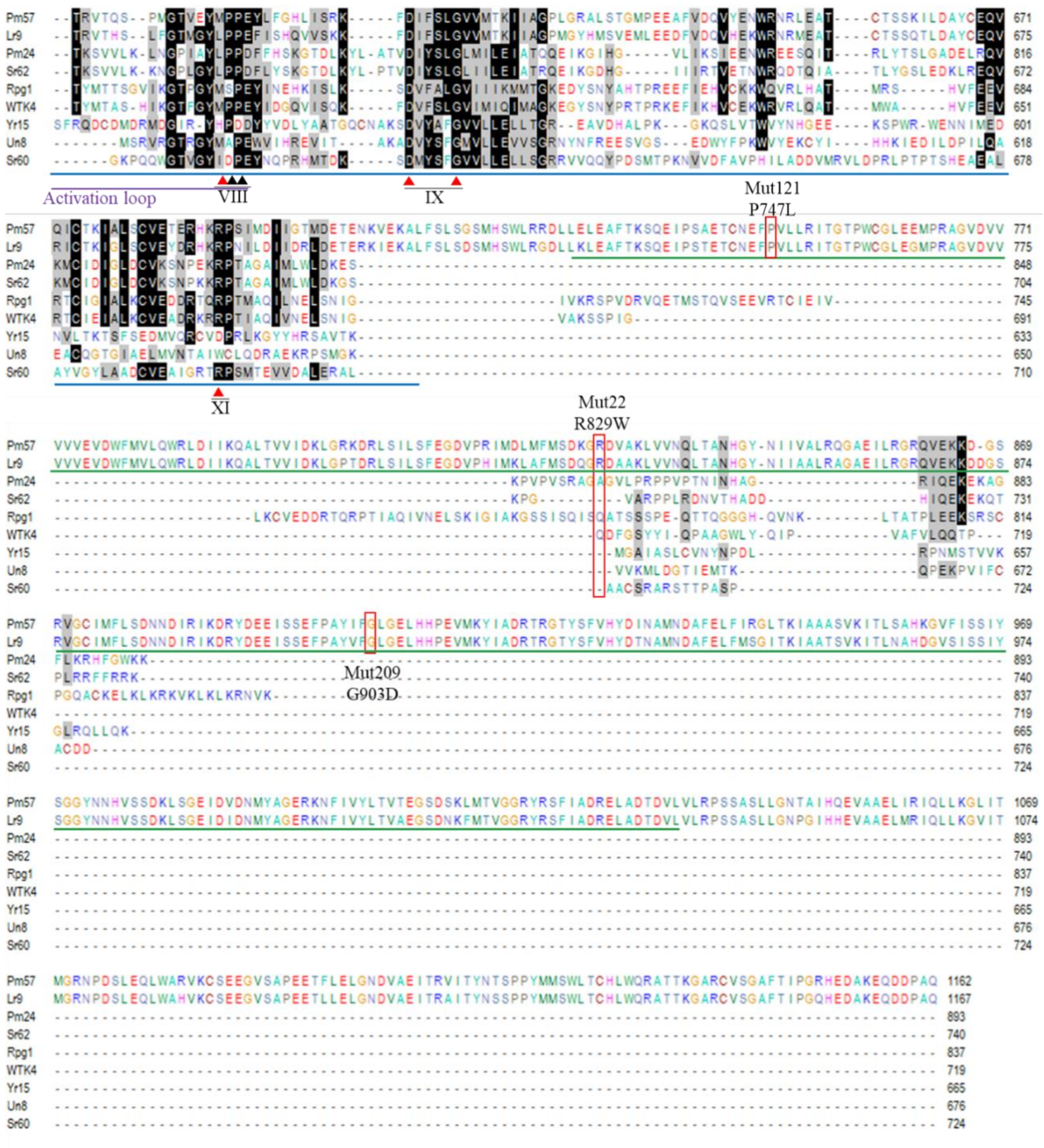

Supplementary Fig. 5. Sequence alignment of WTK Proteins. Based on the sequence conservation of the key amino acid residues in the two kinase domains, the Pm57 Kin I (orange line) is classified as a non-RD kinase and a serine/ threonine protein kinase. The Pm57 Kin II (blue line) is a putative pseudokinase. vWA domain is shown in green line. Black triangles indicate ATP binding site; red triangles indicate key conserved residues; and green triangles positions determining RD or non-RD kinase. The catalytic loop and the activation loop are indicated by light blue and purple colors, respectively. EMS missense mutations that resulted in loss of Pm57 function are indicated by red boxes.

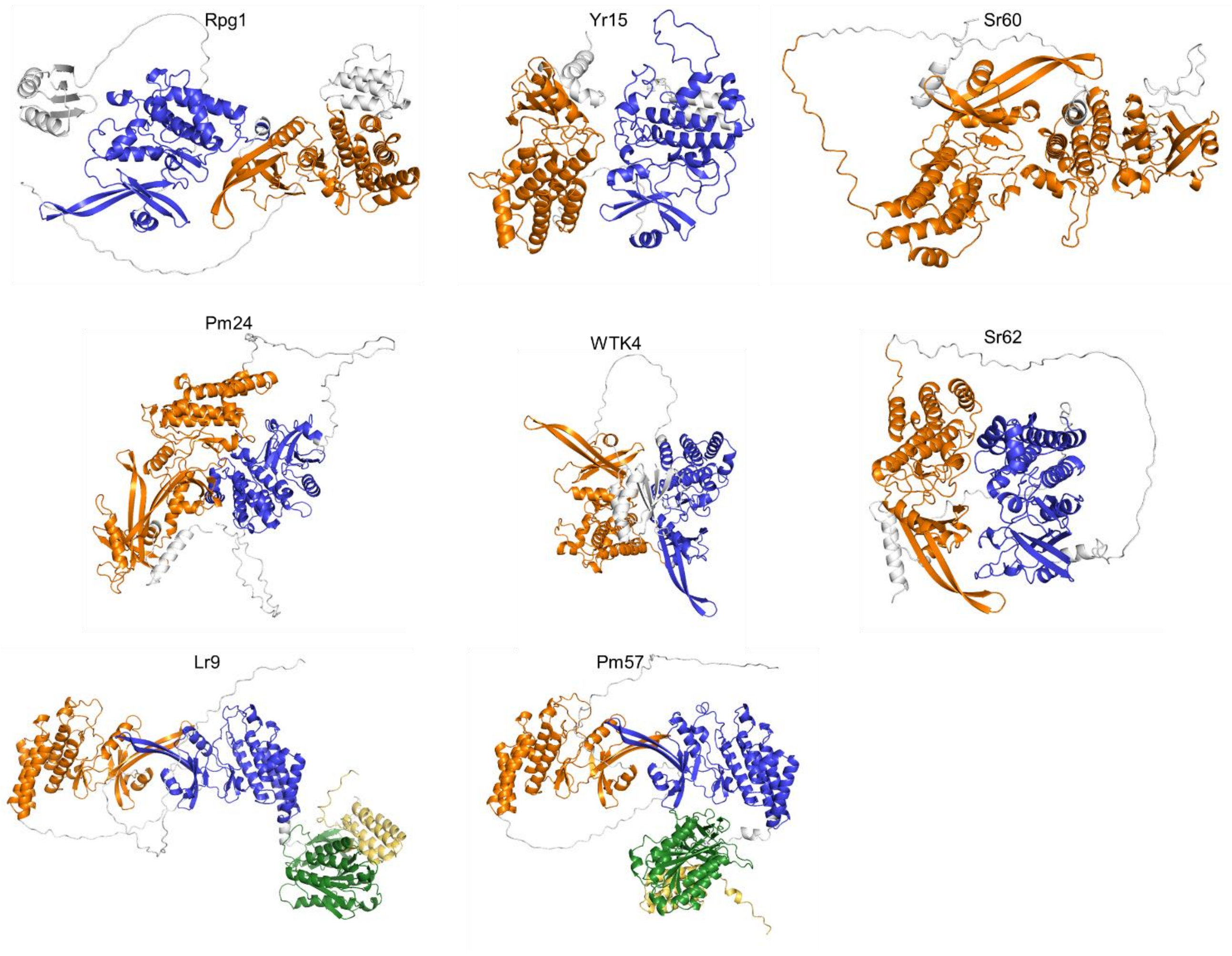

Supplementary Fig. 6. Protein structure prediction of eight cloned WTKs. Three-dimensional model of eight WTKs is predicted by AlphaFold. Orange, kinase domain; blue, pseudokinase domain; Green, vWA domain; Yellow, putative Vwaint domain. The first kinase domain (Kin I) for each protein is shown to the left of that protein structure.

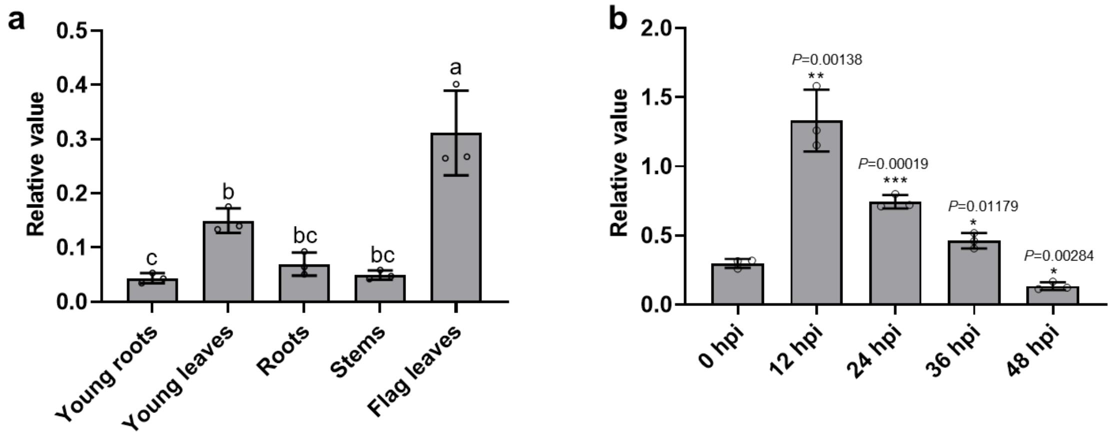

Supplementary Fig. 7. Expression analyses of *Pm57* in 89(5)69. (**a**) Expression levels of *Pm57* in different organs including seedling roots (three-leaf period), seedling leaves (three-leaf period), adult plant roots (heading stage), stems (heading stage) and flag leaves (heading stage). The *TaActin* gene was used as an internal reference. Three biological replicates with tissues from four individual plants mixed as one replicate were used for expression analysis. Data are mean ± SD from three biological replicates. Different lowercase letters above the bars denote significant differences at the $p < 0.05$ level (One-way ANOVA). (**b**) Expression analyses of *Pm57* in *Bgt* E09-inoculated seedlings of 89(5)69. The first leaves of two-leaf seedlings were used to extract total RNA before inoculation (0 h) and 12, 24, 36, and 48 hpi with *Bgt* E09, and for each time point qRT-PCR was performed on three biological replicates with the first leaves from four individual plants mixed as one replicate. *TaActin* was used as endogenous control and the relative expression levels were calculated using the comparative CT method. Data are mean ± SD from three biological replicates. hpi, hours post inoculation. *$p < 0.05$, **$p < 0.01$, ***$p < 0.001$ (two-tailed Student's *t* test). Asterisks indicate significant differences in each time points compared with 0 hpi control. Source data are provided as a Source Data file.

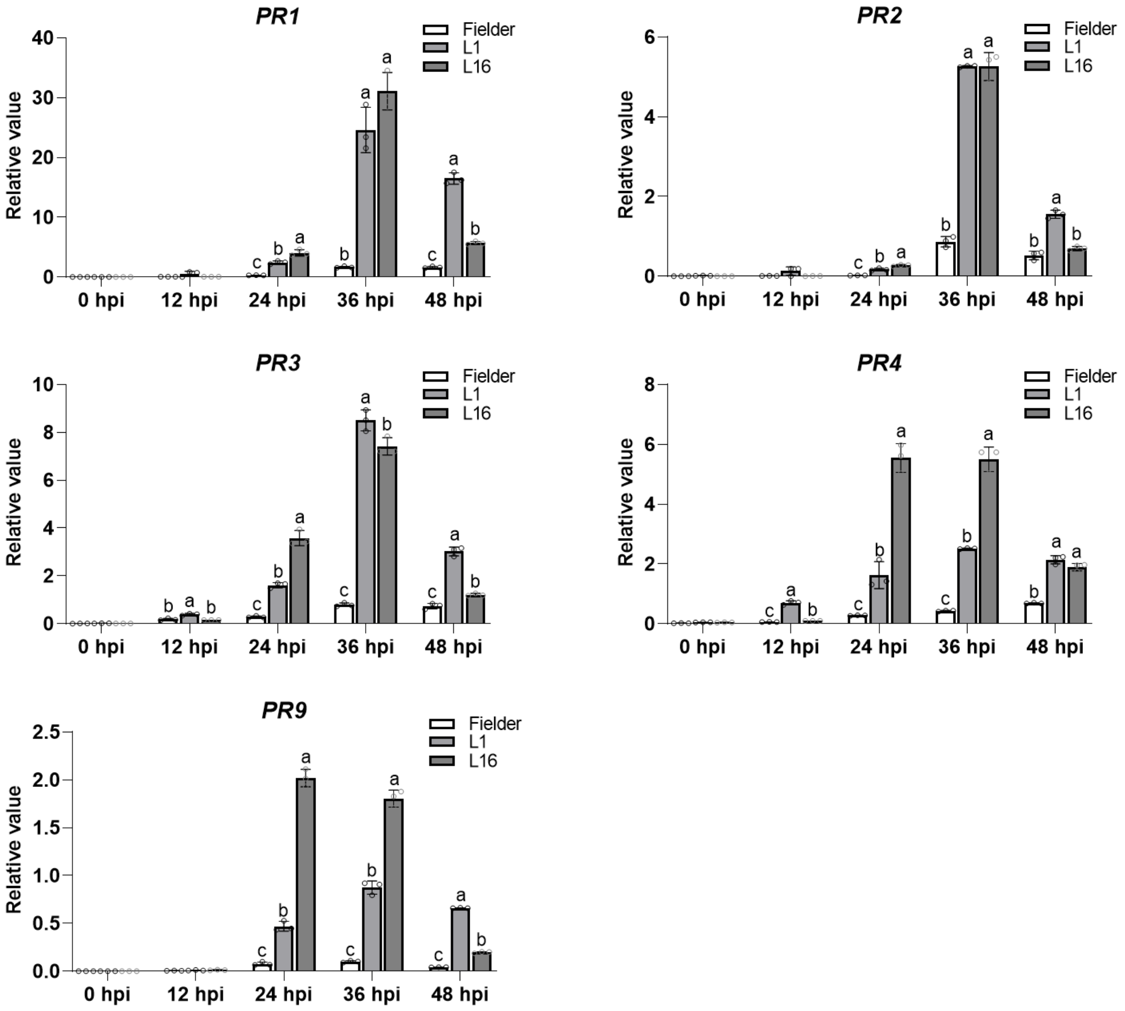

Supplementary Fig. 8. Expression analyses of pathogenesis-related (*PR*) genes in *Pm57* transgenic lines (L1 and L16) and transformation receptor cv Fielder. The first leaves of two-leaf seedlings were used to extract total RNA before inoculation (0 h) and at 12, 24, 36, and 48 hpi with *Bgt* E09, and for each time point qRT-PCR was performed on three biological replicates with the first leaves from four individual plants mixed as one replicate. *TaActin* was used as endogenous control and the transcript levels were calculated using the comparative CT method. Data are mean ± SD from three biological replicates. hpi, hours post inoculation. Different lowercase letters above the bars denote significant differences ($p < 0.05$) of the mean values by One-way ANOVA to differentiate the L1, L16, and Fielder at each time point. Source data are provided as a Source Data file.

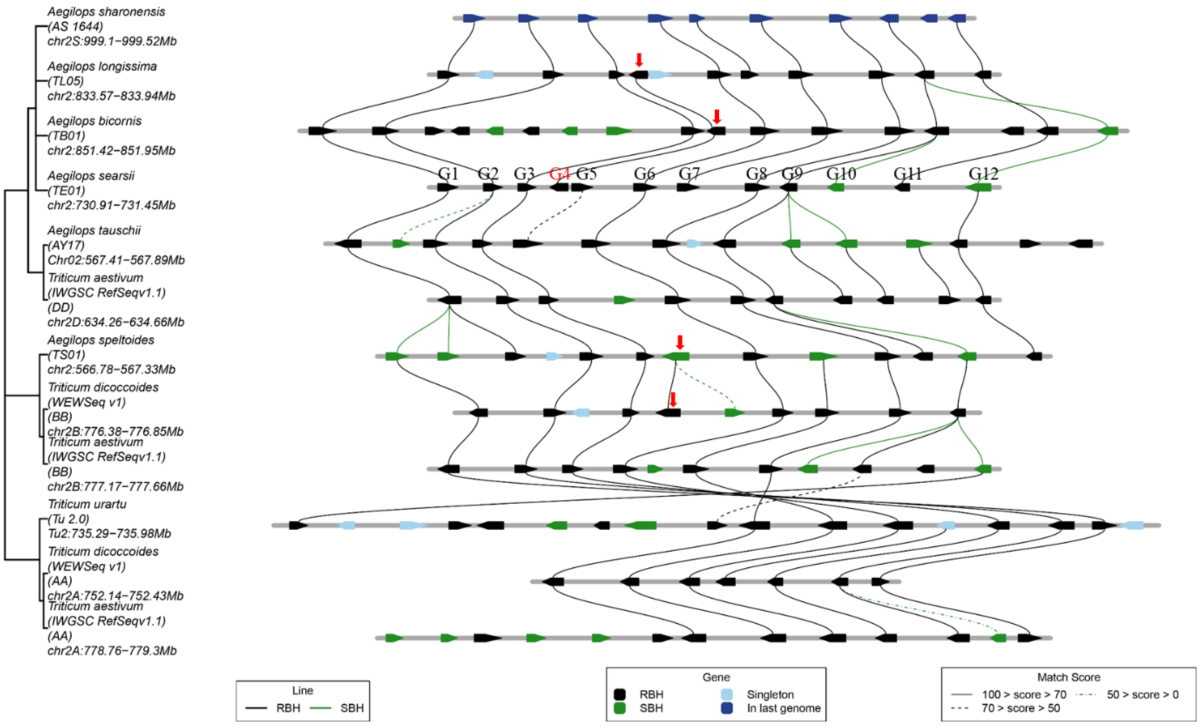

Supplementary Fig. 9. Collinearity of the *Pm57* genomic regions in Triticeae and related species. Collinearity analysis was performed using the online tool Triticeae-GeneTribe[1]. Orthologous genes are linked by lines, and *Pm57* (*G4*) orthologs are shown in red arrows. The *Pm57* orthologs were present only in *Aegilops bicornis* (TB01) (S^bS^b), *Ae. longissima* (TL05) (S^lS^l), *Ae. speltoides* (TS01) (SS), and B sub genome of *T. dicoccoides* (WEWSeq v1) (AABB). RBH (reciprocal best hit), SBH (single-side best hit) and singleton indicated three different types of homologous relationships. RBH: gene pairs belonging to the Reciprocal Best Hits; SBH: gene pairs belonging to the Single-side Best Hits, where RBH is not found but the best matching gene is found; Singleton: the genes with no homologous genes. In last genome: The last genome in the picture. Because the Triticeae-GeneTribe database uses RBH and SBH data structures, where SBH is unidirectional, the last genome cannot be compared with other genomes when drawing from the bottom up. The left tree is obtained from a pre-computed relationship derived from Triticeae-GeneTribe.

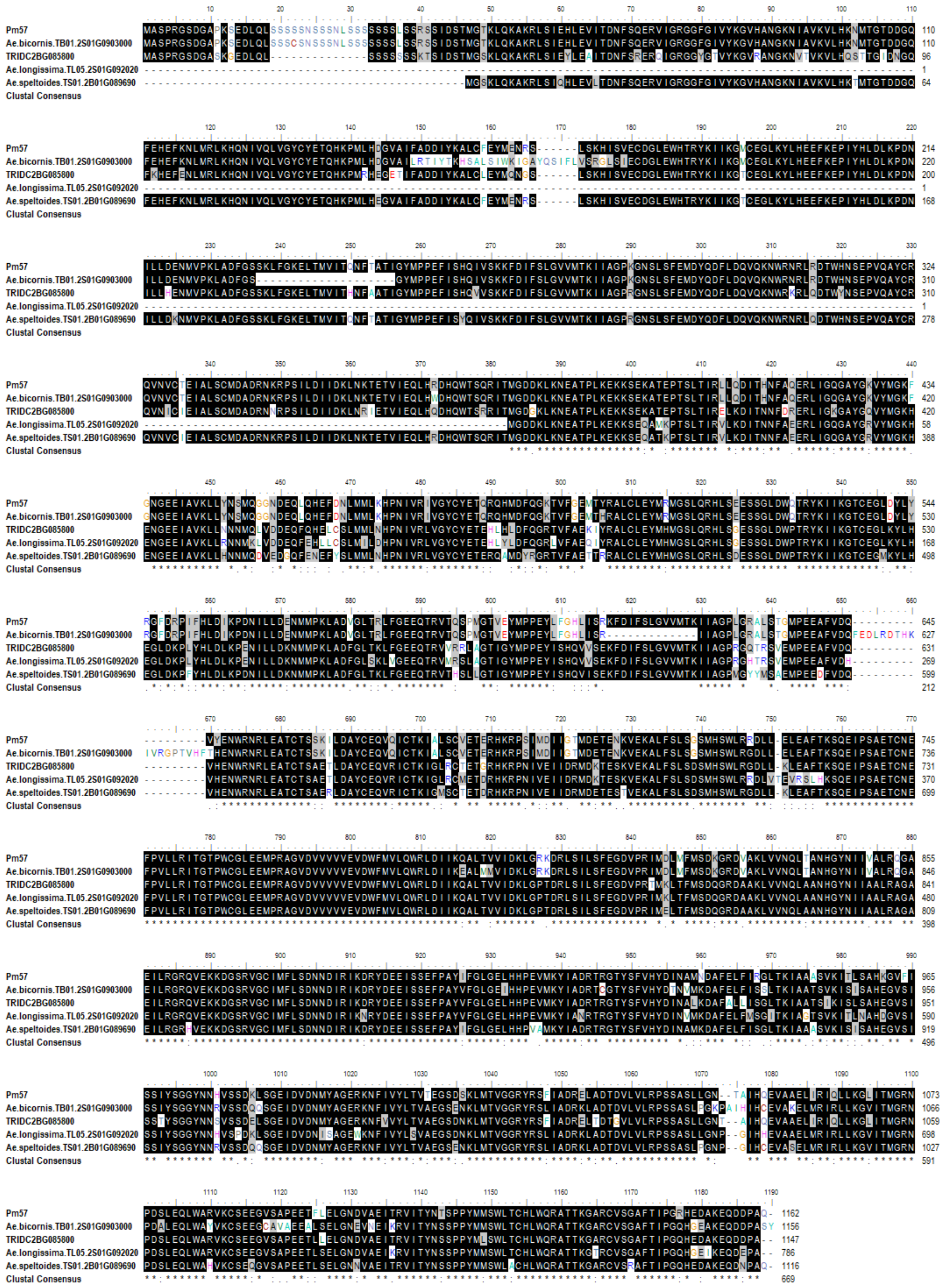

Supplementary Fig. 10. Comparison of amino acid sequences of Pm57 and its orthologs. Amino acid residues conserved in all five sequences are marked by asterisks.

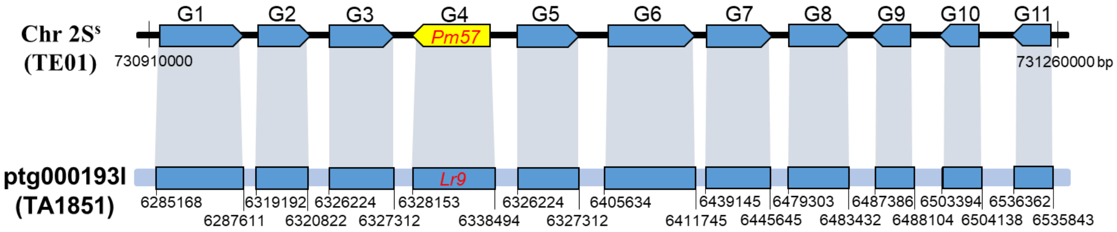

Supplementary Fig. 11. Collinearity of the *Pm57* loci in *Ae. searsii* and *Ae. umbellulata*. The image shows the BLAST results in Supplementary Data 3. The positions of *Lr9* and *Pm57* are indicated.

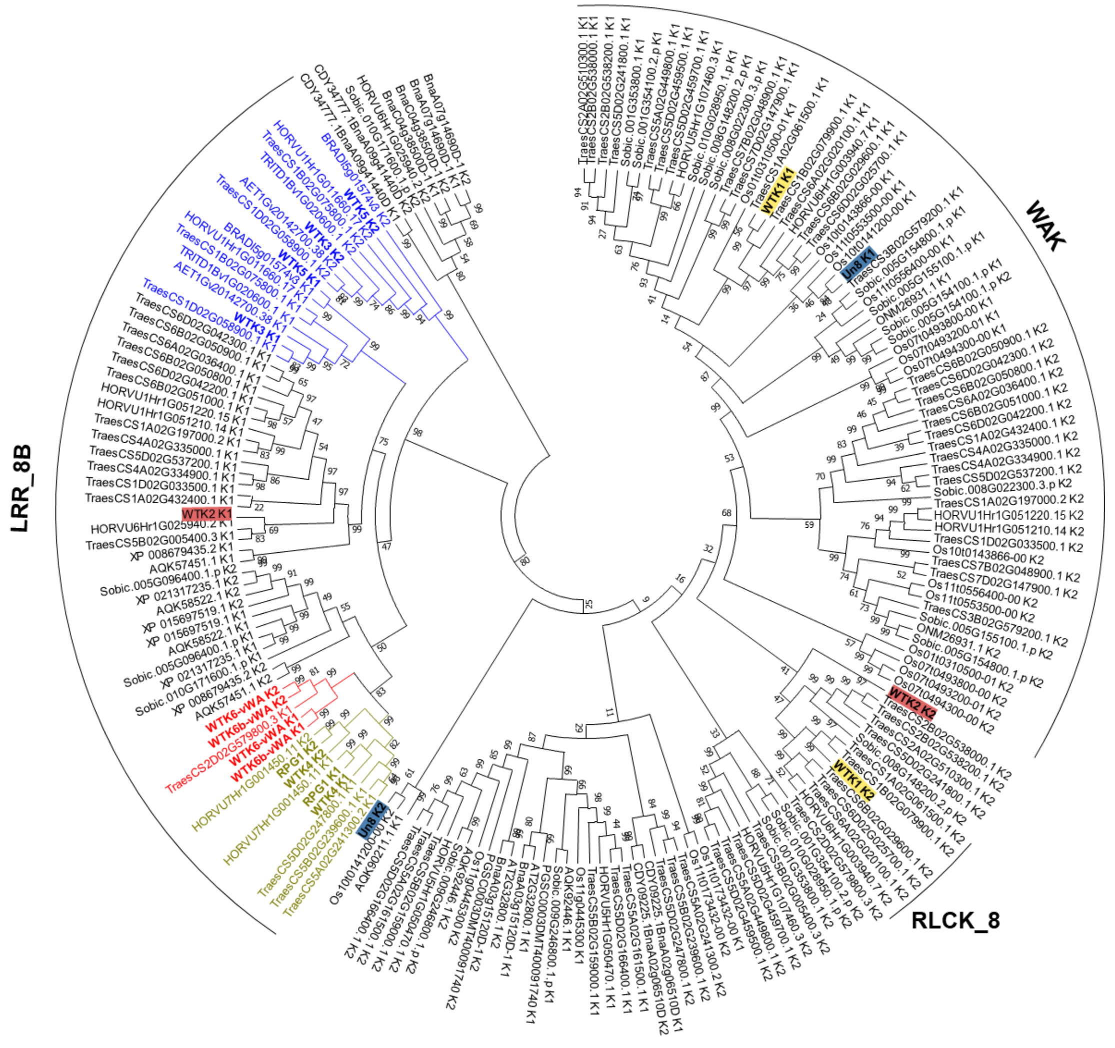

Supplementary Fig. 12. Phylogenetic analysis of plant protein kinase domains. The 182 kinase domains used for phylogenetic analysis of Pm57 were discovered by Klymiuk et al.[2] following the cloning of WTK1. The closest homologs and branches of Kin I (K1) and Kin II (K2) domains of identified WTKs are indicated in different colors. LRR_8B (the kinase domain in cysteine-rich receptor-like kinases), RLCK_8 (receptor-like cytoplasmic kinase subfamily 8), and WAK (cell wall-associated kinases) are three kinase subfamilies[3]. The two kinase domains of Pm57 (WTK6b-vWA) were classified into the same group with two kinase domains of Lr9 (WTK6-vWA) within the LRR_8B subfamily.

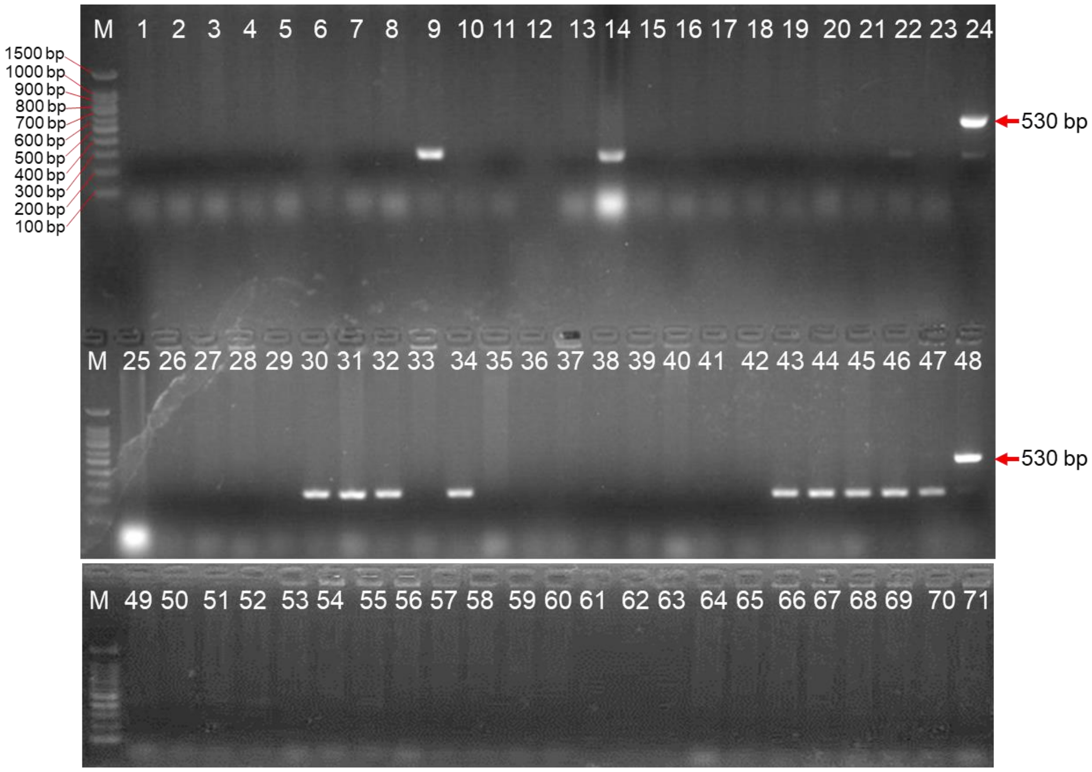

Supplementary Fig. 13. Validation of the functional marker *STS-Pm57*. M, 1 kb ladder; 1-71 was listed in Supplementary Data 4. *Pm57* introgression lines 89(5)69 (Lane 24) and *Ae. searsii* TE01 (Lane 48) as positive controls, and CS (Lane 67) as negative control. Red arrows indicate the amplified fragment of *Pm57* gene using marker *STS-Pm57*.

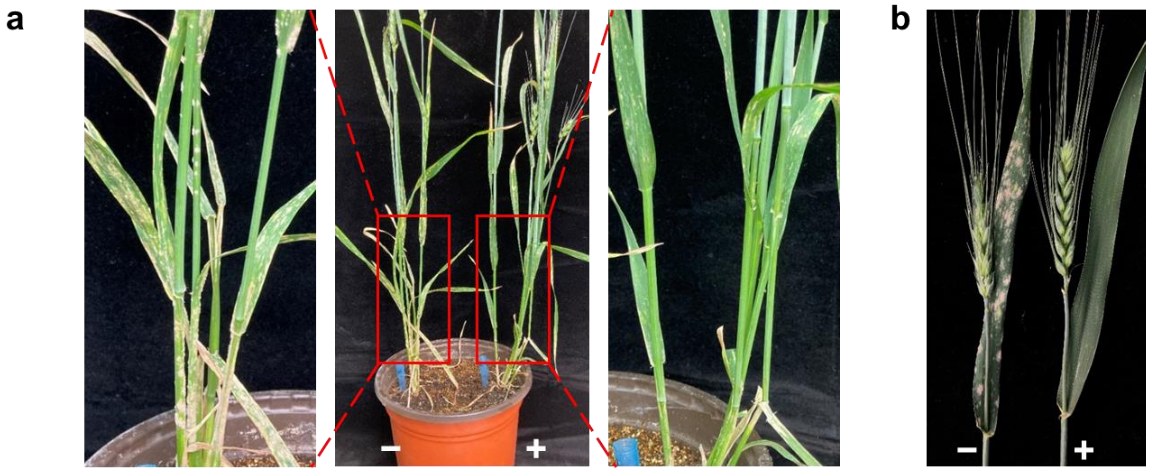

Supplementary Fig. 14. *Pm57* confers an all-stage resistant against powdery mildew. (a-b) Evaluation of the powdery mildew resistance of *Pm57* transgenic plants at adult plant stage in a greenhouse. The '+' and '−' signs on each leaf designate the presence or absence of *Pm57* gene.

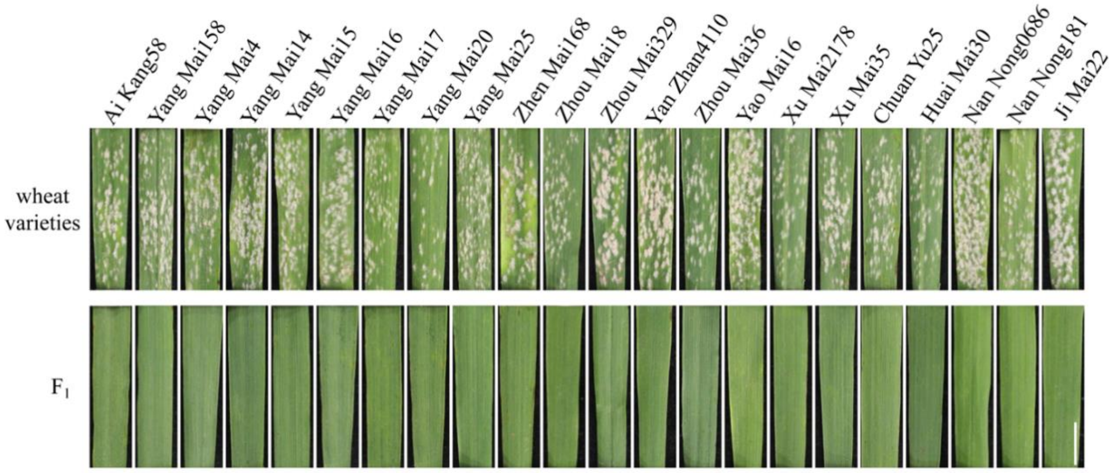

Supplementary Fig. 15. Effect of wheat genetic backgrounds on powdery mildew resistance of *Pm57*. F_1 hybrids were produced by crossing CS-*Ae*. *searsii* introgression line 88R-3-19-1 having small alien segments harboring *Pm57* with 22 wheat varieties as the male parent, respectively. 12 F_1 seedlings, with four seedlings for each of three replicates, were determined for powdery mildew resistance by inoculation with *Bgt* E09. All of the F_1 plants showed high resistance to powdery mildew, indicating *Pm57* confers resistance to powdery mildew in diverse genetic backgrounds of wheat. Bar = 0.5 cm.

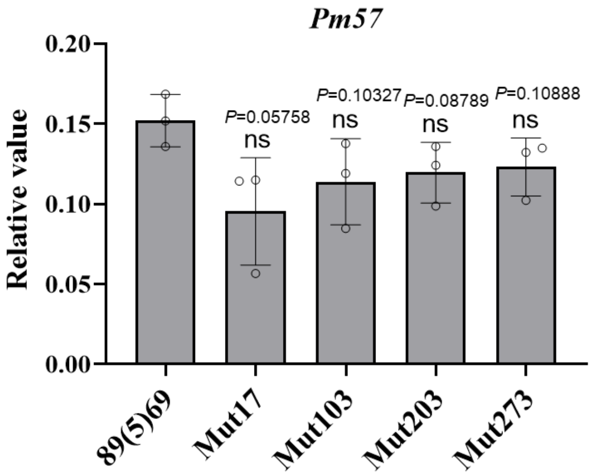

Supplementary Fig. 16. Transcript levels of *Pm57* in the leaves of CS-*Ae. searsii* *Pm57* introgression line 89(5)69 and four EMS-induced susceptible mutants from line 89(5)69. The first leaves of line 89(5)69 and four susceptible mutants at two-leaf stage were respectively sampled to extract RNA. Three biological replicates with leaves from four individual plants mixed as one replicate were used for expression analysis. The wheat *TaActin* was used as an internal reference gene. The transcript levels were calculated using the comparative CT method. Data are mean ± SD from three biological replicates. ns, no statistically significant difference by two-tailed Student's *t* test in each mutant compared with control 89(5)69. Source data are provided as a Source Data file.

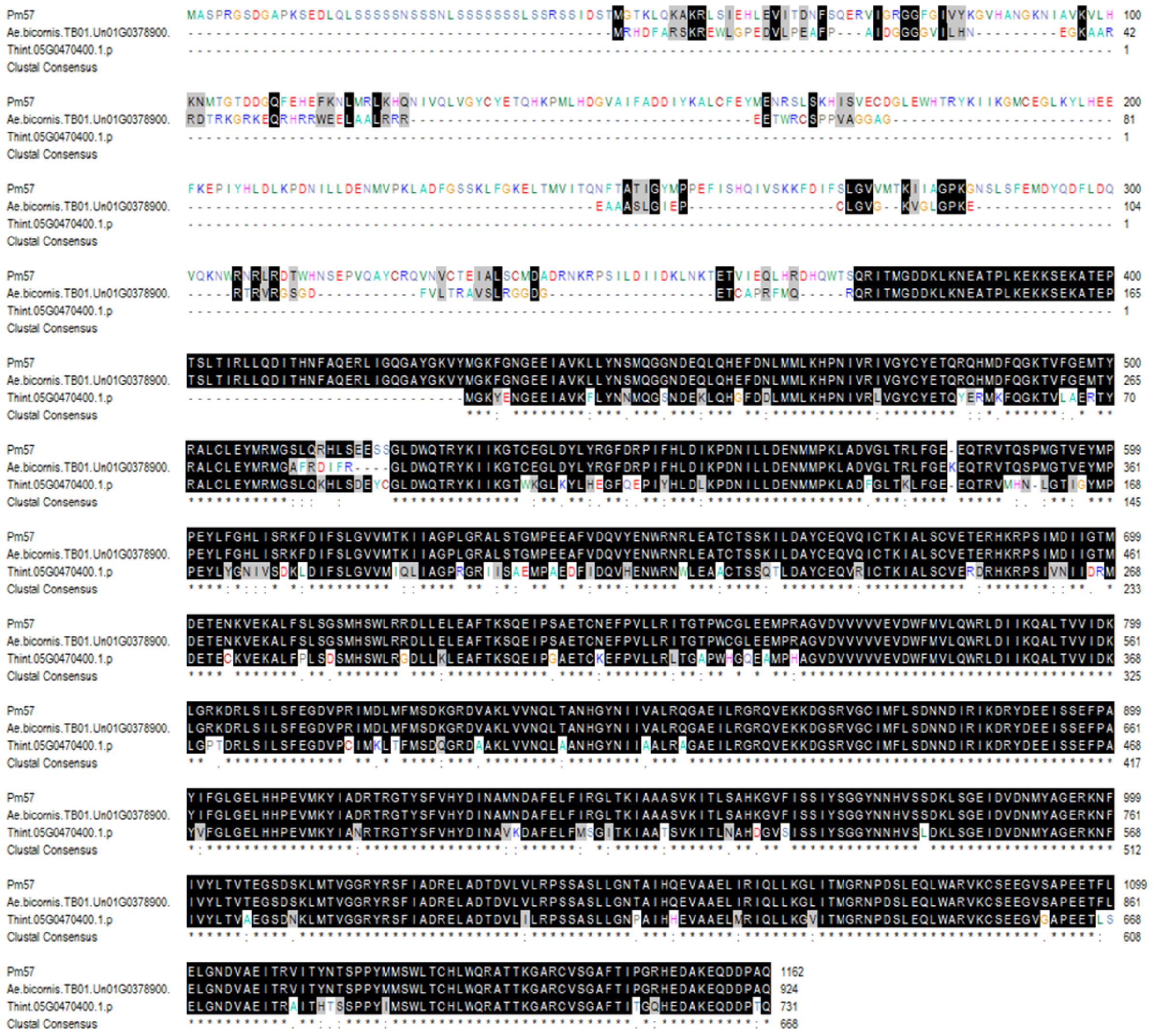

Supplementary Fig. 17. Comparison of amino acid sequences of Pm57 and the other two single kinase-vWA proteins.

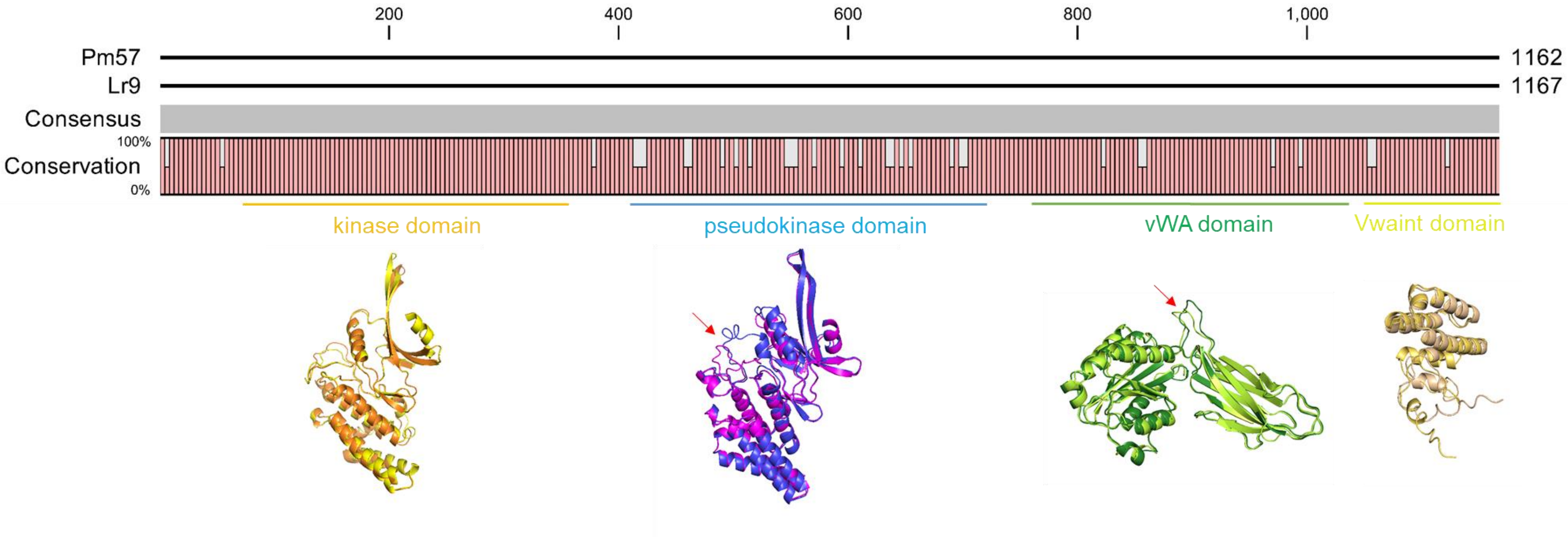

Supplementary Fig. 18. Amino acid sequences alignment and overlap analysis of the predicted protein structures of Pm57 and Lr9. Underlines denotes the location of different structural domains. The sequences of Pm57 and Lr9 were compared using CLC Sequence Viewer 7 software. The 3D protein structures are predicted by AlphaFold. Orange, kinase domain; blue, pseudokinase domain; Green, vWA domain; Yellow, putative Vwaint domain. The red arrows indicate disordered regions.

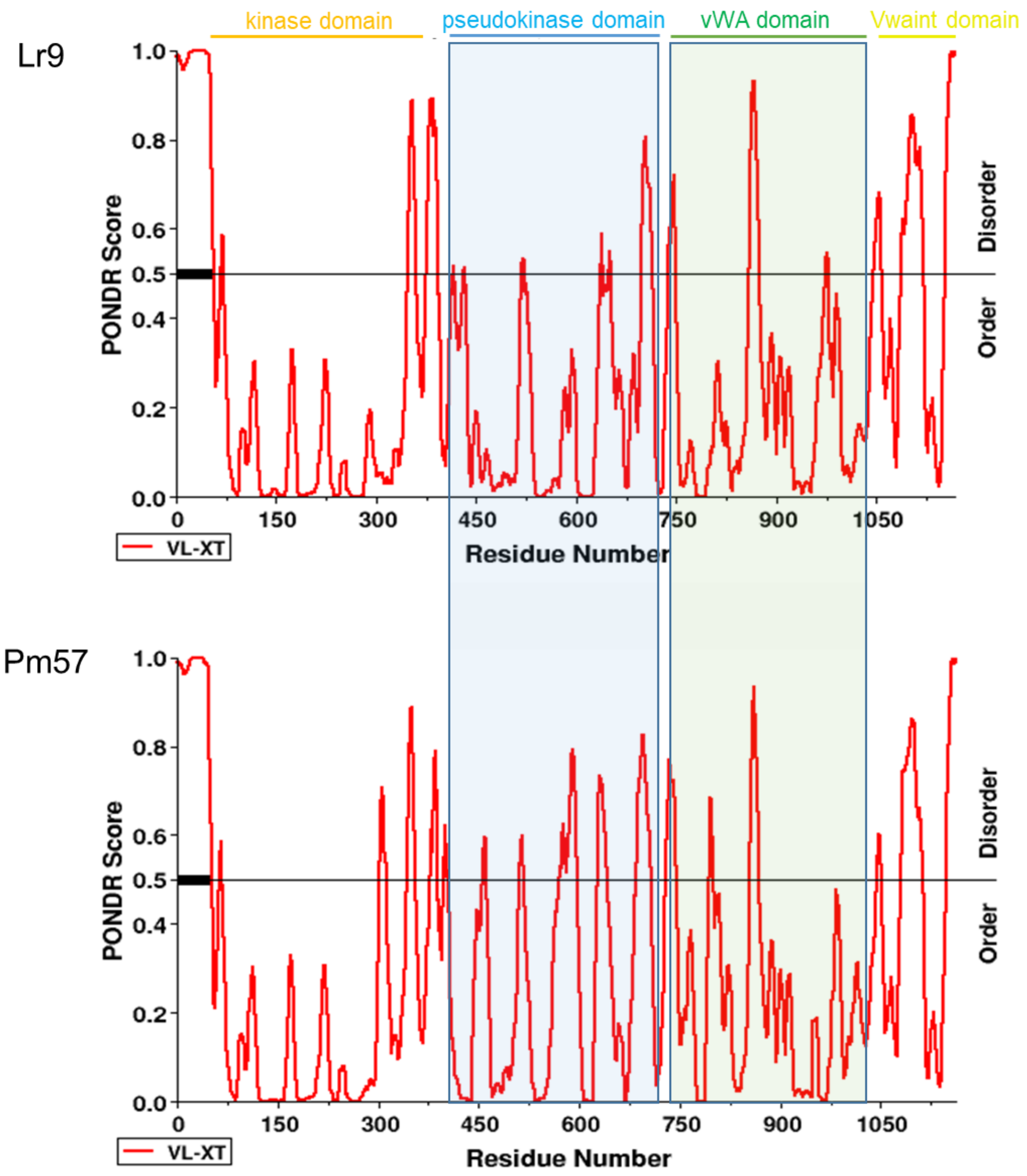

Supplementary Fig. 19. Prediction of the disorder of Lr9 and Pm57 by PONDR analysis. Analysis of the level of residue disorder using VL-XT predictors in PONDR server. Disorder is measured by PONDR score, with values above 0.5 considered to be disordered and values below 0.5 to be ordered. The positions of conservative domains are plotted at the top of the graph, where the pseudokinase and the vWA domains are shaded. In the pseudokinase domain and vWA domain regions, the disorder of Pm57 was higher than that of Lr9.

Supplementary Table 1. BLASTp analysis in Chinese Spring reference genome v1.1 of the twelve annotated genes (G1-G12) in *Pm57* mapping interval of *Ae. searsii* reference genome (TE01).

Name	Query gene (TE01)	Position at chr2S	Description	Subject_gene (CS v1.1)	Query cover	E value	Identity
G1	EVM0036665	730912834-730915214	Glycosyl hydrolase family 3 N terminal domain	TraesCS2B02G591900.2	100%	0	99%
G2	EVM0051586	730937686-730939964	Protein of unknown function (DUF1218)	TraesCS2B02G592000.1	100%	1.49E-129	99%
G3	EVM0012740	730947798-730948886	Isocitrate dehydrogenase NAD subunit	TraesCS2B02G592100.1	100%	0	100%
G4	EVM0016946	730956002-730960059	Tandem kinase protein	-			
G5	EVM0041895	730974837-730979717	Tandem kinase protein	-			
G6	EVM0018635	731043687-731050492	WD40 repeats	TraesCS2B02G592200.1	100%	0	95%
G7	EVM0050643	731061212-731066521	Tyrosine N-monooxygenase	TraesCS2B02G592400.1	111%	0	87%
G8	EVM0056562	731133457-731141575	ETO1-like protein 1	TraesCS2B02G592500.1	100%	0	100%
G9	EVM0002576	731146229-731146967	Dof domain	TraesCS2B02G592600.1	100%	4.27E-124	85%
G10	EVM0023928	731172579-731173314	Dof domain	TraesCS2B02G592700.1	104%	9.45E-146	92%
G11	EVM0051934	731258724-731259457	Dof domain	TraesCS2B02G592800.1	97%	2.59E-104	80%
G12	EVM0049887	731392074-731453412	Dof domain	TraesCS2B02G592900.1	100%	1.52E-126	88%

The WheatOmics 1.0 (http://202.194.139.32/blast/blast.html) was used for BLAST analysis with default parameters.

Supplementary Table 2. Read counts of the twelve genes in the *Pm57* mapping interval in susceptible mutants and the parental line 89(5)69 carrying *Pm57*.

Name	ID	89(5)69	Mut51	Mut60	Mut141	Mut209	Mut216
G1	EVM0036665	0	0	0	0	0	0
G2	EVM0051586	1312	2729	2058	1966	2271	785
G3	EVM0012740	0	0	0	1	0	0
G4	EVM0016946	2312	1946	589	1083	1347	1414
G5	EVM0041895	321	380	177	324	200	108
G6	EVM0018635	13	7	30	77	470	6
G7	EVM0050643	1312	1553	98	322	477	716
G8	EVM0056562	2248	3289	1185	2462	2366	1559
G9	EVM0002576	21	382	960	180	374	223
G10	EVM0023928	30	520	811	76	582	119
G11	EVM0051934	36	175	45	92	116	24
G12	EVM0049887	2	41	211	19	54	24

Supplementary Table 3. Powdery mildew resistance evaluation of the T_1 transgenic plants to *Bgt* isolate E09.

T_1 transgenic family	Trangenic positive plants			Trangenic negative plants	
	No. of positive plants	No. of resistant plants	No. of susceptible plants	No. of negative plants	No. of susceptible plants
L1	11	11	0	1	1
L2	12	12	0	0	0
L3	9	9	0	3	3
L4	8	8	0	4	4
L5	0	0	0	12	12
L6	12	12	0	0	0
L7	8	8	0	4	4
L8	9	9	0	3	3
L9	11	11	0	0	0
L10	10	10	0	1	1
L11	10	10	0	1	1
L12	8	8	0	4	4
L13	9	9	0	2	2
L14	9	9	0	3	3
L15	11	11	0	0	0
L16	12	12	0	0	0
L17	9	9	0	2	2
L18	9	9	0	3	3
L19	11	11	0	0	0
L20	10	0	10	1	1
L21	11	11	0	1	1
L22	12	12	0	0	0
L23	0	0	0	12	12
L24	9	9	0	2	2
L25	10	10	0	2	2
L26	8	8	0	2	2
L27	0	0	0	11	11
L28	9	9	0	3	3
L29	12	0	12	0	0
L30	10	10	0	2	2
L31	12	12	0	0	0
L32	11	11	0	0	0
L33	8	8	0	4	4
L34	12	12	0	0	0
L35	10	10	0	2	2
L36	9	9	0	3	3

Supplementary Table 4. The results of *Lr9*-vs-*Pm57* reciprocal BLAST between *Ae. searsii* (TE01) and *Ae. umbellulata* (TA1851) assemblies.

Query	Subject	Identity	Alignment length	Gaps	Query_start	Query_end	Subject_start	Subject_end	E value
Lr9 genomic sequences	Ae.searsii.TE01 chr2S	93.5%	5489	158	1	5489	730965667	730960179	0
Lr9 genomic sequences	Ae.searsii.TE01 chr2S	85.3%	2412	81	6277	8688	730960274	730957863	0
Lr9 genomic sequences	Ae.searsii.TE01 chr2S	93.7%	1066	5	9008	10073	730957255	730956190	0
Pm57 genomic sequences	ptg000193l	95.3%	4314	24	12	4275	6338494	6334189	0
Pm57 genomic sequences	ptg000193l	91.2%	1285	34	4263	5502	6334243	6332980	0
Pm57 genomic sequences	ptg000193l	90.5%	401	4	5590	5990	6332058	6331664	2.57E-145
Pm57 genomic sequences	ptg000193l	86.5%	1887	14	6025	7902	6331656	6329788	0
Pm57 genomic sequences	ptg000193l	92.1%	1266	10	8517	9776	6329394	6328153	0

BLAST analysis was performed by BLASTN 2.13.0+ software with default settings. The best BLAST hits with the *Lr9* genomic sequences was *Pm57* of *Ae. searsii* (TE01) and the best BLAST hits with the *Pm57* genomic sequences was *Lr9* of *Ae. umbellulata* (TA1851).

Supplementary Table 5. Analysis of *Ka* and *Ks* ratio of *Pm57* and *Lr9* genes.

**Sequence**	***Ka***	***Ks***	***Ka/Ks***	***P*-value (Fisher)**	**Length**	**Substitutions**	**Syn-Subs**	**Nonsyn-Subs**	**Divergence-Distance**
Pm57-*Lr9*	0.061805	0.147588	0.418769	3.20E-12	3480	261	107.802	153.198	0.08133
*Pm57*K1-*Lr9*K1	0.008878	0.064196	0.13829	2.33E-05	927	19	12.9346	6.06541	0.021476
*Pm57*K2-*Lr9*K2	0.161503	0.209447	0.771096	0.144977	918	136	36.0216	99.9784	0.171927
*Pm57*vWA-*Lr9*vWA	0.045543	0.242153	0.188075	2.73E-13	927	69	40.2467	28.7533	0.086515

K1: kinase domain; K2: pseudokinase domain. The *Ka* (non-synonymous substitution) and *Ks* (synonymous substitution) substitution rates was calculated using KaKs_Calculator 3.0. *P*-Value (Fisher): The value computed by two-sided Fisher exact test.

Supplementary Table 6. Infection type of CS-*Ae. searsii Pm57* introgression line 89(5)69 and *Pm57* transgenic plants to divergent *Bgt* isolates.

No.	*Bgt*	Collection site	CS	89(5)69	Fielder	Transgenic plants
1	GY-KDZ-1	Gongyi, Henan	4	0	4	0
2	HB-XX-WXZ-1	Hebi, Henan	3	0	4	0
3	SQ-YC-YJZ	Shangqiu, Henan	3	0	4	0
4	ZK-CHQ-LHZ-2	Zhoukou, Henan	4	0	4	0
5	SL-FS-5	Pingdingshan,Henan	4	0	4	0
6	SQ-YC-CGS	Shangqiu, Henan	3	0	4	0
7	ZK-HY-QLZ-1	Zhoukou, Henan	4	0;	4	0
8	ZK-HY-QLZ-2	Zhoukou, Henan	3	0	4	0
9	KF-YLH	Kaifeng, Henan	4	0	4	0
10	Y01		4	0	4	0
11	Y02		4	0	4	0
12	Y03		3	1	4	0
13	Y04		4	0	4	0
14	Y06		4	0	4	0
15	Y07		4	0	4	0
16	Y08		4	0	4	0
17	Y09		4	0	4	0
18	Y10		4	0	4	0
19	Y11		4	0	4	0
20	Y14		4	0;	4	0
21	Y15		4	0	4	0
22	Y16		4	0	4	0
23	Y17		4	0	4	0
24	Y18		3	0	4	0
25	Y21		4	0	4	0
26	B18		4	0	4	0
27	E05	Yunnan province	4	0	4	0
28	E09	Beijing	4	0	4	0
29	E26	Guizhou province	4	0	4	0

The first 9 *Bgt* isolates in the list were provided by Prof. Wenming Zheng from Henan Agricultural University, and the remaining 20 single-spore derived *Bgt* isolates with different virulent spectrum were provided by Prof. Pengtao Ma from Yantai University. Infection types 0-1 were resistant and 3-4 were susceptible.

Supplementary Table 7. List of plant materials used for *Pm57* mapping and cloning in this study.

No.	Accession no.	Label	Description
1	TA3808	CS	Chinese Spring, for *Pm57* mapping
2	TA3809		CS *ph1b* mutant, for *Pm57* mapping
3	TA3581		CS-*Ae. searsii* disomic $2S^s$ addition line, for *Pm57* mapping
4	TA5109	89(5)69	CS-*Ae. searsii* T2BS.2BL-$2S^sL$ recombinant line, for *Pm57* cloning and expression analyses
5		89(6)88	CS-*Ae. searsii* Ti2AS-$2S^sS.2S^sL$-2AL recombinant line, for *Pm57* mapping
6	TE01	TE01	*Ae. searsii*, for *Pm57* cloning
7	CItr 17268	Fielder	Common wheat, for wheat protoplast preparation and genetic transformation
8		88R-3-19-1	CS-*Ae. searsii Pm57* recombinant with the shortest $2S^s$ genomic region was found in a previous study[34]
9		Ai Kang58, Yang Mai158, Yang Mai4, Yang Mai14, Yang Mai15, Yang Mai16, Yang Mai17, Yang Mai20, Yang Mai25, Zhen Mai168, Zhou Mai18, Zhou Mai329, Yan Zhan4110, Zhou Mai36, Yao Mai16, Xu Mai2178, Xu Mai35, Chuan Yu25, Huai Mai30, Nan Nong0686, Nan Nong181, Ji Mai22	22 wheat varieties were crossed with 88R-3-19-1 to check whether *Pm57* can confer powdery mildew resistance in diverse genetic backgrounds

Supplementary references

1. Chen, Y. *et al*. A collinearity-incorporating homology inference strategy for connecting emerging assemblies in the Triticeae tribe as a pilot practice in the plant pangenomic era. *Mol Plant* **13**, 1694-1708 (2020).
2. Klymiuk, V. *et al*. Cloning of the wheat *Yr15* resistance gene sheds light on the plant tandem kinase-pseudokinase family. *Nat. Commun.* **9**, 3735 (2018).
3. Zulawski, M., Schulze, G., Braginets, R., Hartmann, S. & Schulze, W.X. The *Arabidopsis* Kinome: phylogeny and evolutionary insights into functional diversification. *BMC Genomics* **15**, 548 (2014).
