# Supplementary material for: Pm57 from Aegilops searsii encodes a tandem kinase protein and confers wheat powdery mildew resistance
Source: Nat Commun. 2024 Jun 5;15:4796. doi: 10.1038/s41467-024-49257-2 (PMC11153570; doi:10.1038/s41467-024-49257-2)
Supplement: Supplementary file 3 — Description of Additional Supplementary Files [file 41467_2024_49257_MOESM3_ESM.pdf]

Description of Additional Supplementary Files

File Name: Supplementary Data 1
Description: Primers used in this study.

File Name: Supplementary Data 2
Description: The proteins with the tandem kinase -vWA domains or a single kinase domain followed by a vWA domain in plants.

File Name: Supplementary Data 3
Description: BLASTn analysis in *Ae. umbellulata* TA1851 assemblies of the twelve annotated genes (G1-G12) in *Pm57* mapping interval of *Ae. searsii* reference genome (TE01).

File Name: Supplementary Data 4
Description: List of plant materials used to check the presence of the *Pm57* resistance allele.
