# Supplementary material for: Pm57 from Aegilops searsii encodes a tandem kinase protein and confers wheat powdery mildew resistance
Source: Nat Commun. 2024 Jun 5;15:4796. doi: 10.1038/s41467-024-49257-2 (PMC11153570; doi:10.1038/s41467-024-49257-2)
Supplement: Supplementary file 8 — Reporting Summary [file 41467_2024_49257_MOESM8_ESM.pdf]

nature portfolio

Corresponding author(s): Wenxuan Liu

Last updated by author(s): May 27, 2024

Reporting Summary

Nature Portfolio wishes to improve the reproducibility of the work that we publish. This form provides structure for consistency and transparency in reporting. For further information on Nature Portfolio policies, see our Editorial Policies and the Editorial Policy Checklist.

Statistics

For all statistical analyses, confirm that the following items are present in the figure legend, table legend, main text, or Methods section.

n/a	Confirmed	
☐	☒	The exact sample size (*n*) for each experimental group/condition, given as a discrete number and unit of measurement
☐	☒	A statement on whether measurements were taken from distinct samples or whether the same sample was measured repeatedly
☐	☒	The statistical test(s) used AND whether they are one- or two-sided<br>*Only common tests should be described solely by name; describe more complex techniques in the Methods section.*
☒	☐	A description of all covariates tested
☐	☒	A description of any assumptions or corrections, such as tests of normality and adjustment for multiple comparisons
☐	☒	A full description of the statistical parameters including central tendency (e.g. means) or other basic estimates (e.g. regression coefficient) AND variation (e.g. standard deviation) or associated estimates of uncertainty (e.g. confidence intervals)
☐	☒	For null hypothesis testing, the test statistic (e.g. *F*, *t*, *r*) with confidence intervals, effect sizes, degrees of freedom and *P* value noted<br>*Give P values as exact values whenever suitable.*
☒	☐	For Bayesian analysis, information on the choice of priors and Markov chain Monte Carlo settings
☒	☐	For hierarchical and complex designs, identification of the appropriate level for tests and full reporting of outcomes
☒	☐	Estimates of effect sizes (e.g. Cohen's *d*, Pearson's *r*), indicating how they were calculated

Our web collection on statistics for biologists contains articles on many of the points above.

Software and code

Policy information about availability of computer code

Data collection	n/a
Data analysis	1. DNAMAN 7 software<br>2. Microsoft excel 2021<br>3. HISAT2 v2.0.5<br>4. SAMtools v1.9<br>5. featureCounts tool v1.4.4<br>6. BCFtools v1.7<br>7. AlphaFold v2.1<br>8. PyMOL v. 2.6.0a0<br>9. MEGA7.0<br>10. BLASTN 2.13.0+ software<br>11.KaKs_Calculator 3.0<br>12. Triticeae-GeneTribe (http://wheat.cau.edu.cn/TGT/)<br>13. Predictor of Naturally Disordered Regions (PONDR; http://www.pondr.com/)

For manuscripts utilizing custom algorithms or software that are central to the research but not yet described in published literature, software must be made available to editors and reviewers. We strongly encourage code deposition in a community repository (e.g. GitHub). See the Nature Portfolio guidelines for submitting code & software for further information.

Data

Policy information about availability of data

All manuscripts must include a data availability statement. This statement should provide the following information, where applicable:

- Accession codes, unique identifiers, or web links for publicly available datasets
- A description of any restrictions on data availability
- For clinical datasets or third party data, please ensure that the statement adheres to our policy

Data supporting the findings of this work are available within the paper and its Supplementary Information files. Detailed sequence data of Pm57 gene was deposited in NCBI database under accession OQ675542 (https://www.ncbi.nlm.nih.gov/nuccore/OQ675542.1/). The MutRNA-Seq data derived from the WT and five mutant plants have been deposited in NCBI's Sequence Read Archive (SRA) under accession number PRJNA947672 (https://www.ncbi.nlm.nih.gov/bioproject/PRJNA947672/). The following public databases were used in this study: WheatOmics 1.0 (http://wheatomics.sdau.edu.cn/), and Phytozome v13 database (https://phytozome-next.jgi.doe.gov/). Source data are provided with this paper.

Research involving human participants, their data, or biological material

Policy information about studies with human participants or human data. See also policy information about sex, gender (identity/presentation), and sexual orientation and race, ethnicity and racism.

Reporting on sex and gender	n/a
Reporting on race, ethnicity, or other socially relevant groupings	n/a
Population characteristics	n/a
Recruitment	n/a
Ethics oversight	n/a

Note that full information on the approval of the study protocol must also be provided in the manuscript.

Field-specific reporting

Please select the one below that is the best fit for your research. If you are not sure, read the appropriate sections before making your selection.

[x] Life sciences [ ] Behavioural & social sciences [ ] Ecological, evolutionary & environmental sciences

For a reference copy of the document with all sections, see nature.com/documents/nr-reporting-summary-flat.pdf

Life sciences study design

All studies must disclose on these points even when the disclosure is negative.

Sample size	A total of 3,380 F3 individuals were screened using two Pm57 flanking markers, resulting in the identification of 104 CS-Ae. searsii 2Ss recombinants. By integrating the Bgt responses of the 104 recombinants with the 16-marker analysis, we mapped Pm57 to the region between markers X10 and X13. This region corresponds to a physical interval of 710 kb on the long arm of Ae. searsii chromosome 2Ss, which harbours 12 genes.
Data exclusions	We did not exclude any data points arbitrarily.
Replication	All attempts at replication were successful. The number of replications is indicated in the corresponding figure legend and/or in the corresponding material and method section.
Randomization	All samples from each experiments were collected randomly for further analysis.
Blinding	All investigations were blinded to group allocation during data collection and analysis.

Reporting for specific materials, systems and methods

We require information from authors about some types of materials, experimental systems and methods used in many studies. Here, indicate whether each material, system or method listed is relevant to your study. If you are not sure if a list item applies to your research, read the appropriate section before selecting a response.

Materials & experimental systems

n/a	Involved in the study
☒	☐ Antibodies
☒	☐ Eukaryotic cell lines
☒	☐ Palaeontology and archaeology
☒	☐ Animals and other organisms
☒	☐ Clinical data
☒	☐ Dual use research of concern
☐	☒ Plants

Methods

n/a	Involved in the study
☒	☐ ChIP-seq
☒	☐ Flow cytometry
☒	☐ MRI-based neuroimaging

Plants

Seed stocks	All seeds used in this study were stored in our lab.
Novel plant genotypes	In this study, we obtained the Pm57 transgenic lines in wheat plant.
Authentication	The specific PCR primers 3F/2R were used to identify the T0 and T1 Fielder transgenic progenies.
